# The Brazilian version of the High-Activity Arthroplasty Score: psychometric property evaluation in hip replacement patients

**DOI:** 10.1590/1516-3180.2024.0176.27112025

**Published:** 2025-08-04

**Authors:** Nathalia Sundin Palmeira de Oliveira, Julia Ribeiro Soares, Danúbia da Cunha de Sá Caputo, Gustavo Leporace, Luiz Alberto Batista, Themis Moura Cardinot, Liszt Palmeira de Oliveira

**Affiliations:** I Postgraduate Program in Medical Sciences, Universidade do Estado do Rio de Janeiro (UERJ), Rio de Janeiro (RJ), Brazil.; II Faculdade de Ciências Médicas (FCM), Universidade do Estado do Rio de Janeiro (UERJ), Rio de Janeiro (RJ), Brazil.; III Department of Biophysics and Biometrics, Universidade do Estado do Rio de Janeiro (UERJ), Rio de Janeiro (RJ), Brazil.; IV Professor, Department of Diagnostic Imaging, Universidade Federal de São Paulo (UNIFESP), São Paulo (SP), Brazil; Chief of Biomechanics Research, Instituto Brasil de Tecnologias de Saúde (IBTS), Rio de Janeiro (RJ), Brazil.; V Professor, Institute of Physical Education and Sports, Universidade do Estado do Rio de Janeiro (UERJ), Rio de Janeiro (RJ), Brazil.; VI Professor, Department of Pharmaceutical Sciences, Universidade Federal Rural do Rio de Janeiro (UFRRJ), Rio de Janeiro (RJ), Brazil.; VII Professor, Department of Surgical Specialties, Universidade do Estado do Rio de Janeiro (UERJ), Rio de Janeiro (RJ), Brazil.

**Keywords:** Arthroplasty, replacement, hip, Reproducibility of results, Validation study, Patient-reported outcome measure, Sports, Outcome measurement, Reliability and validity, Physical activity, Exercise

## Abstract

**BACKGROUND:**

The High-Activity Arthroplasty Score (HAAS) is a reliable and valid self-administered questionnaire that was developed in British English and was designed to determine the level of physical activity in patients after lower limb arthroplasty (hip and/or knee). The Brazilian version (HAAS-Brazil) was developed after a cross-cultural adaptation in 2023.

**OBJECTIVE:**

To evaluate the psychometric properties of HAAS-Brazil in patients after hip arthroplasty.

**DESIGN AND SETTING:**

A cross-sectional quantitative and qualitative study was conducted in an orthopedic outpatient setting.

**METHODS:**

Evidence for the validity of HAAS-Brazil was assessed via psychometric testing, which followed the COnsensus-based Standards for the selection of health status Measurement INstruments (COSMIN).

**RESULTS:**

A total of 112 patients with a mean age of 56 years were included as participants; of these patients, 50.9% were female, with 44.6% being overweight and 85.7% being engaged in physical activity. HAAS-Brazil provided satisfactory evidence of content validity (CVC > 0.9), structural validity (AISP = 1; H_i_ > 0.3; VI_Mon_ = 0; VI_IIO_ = 0), construct validity (**ρ**_HOS-SP_ = 0.696; **ρ**_SF-12 PSC_ = 0.554; **ρ**_SF-1 MSC_ = 0.338), no ceiling or floor effect, acceptable internal consistency (Mokken **ρ** = 0.707; Cronbach **α** = 0.663), and good reliability (ICC_(3,K)_ = 0.840 ; P < 0.001).

**CONCLUSION:**

The HAAS-Brazil provided satisfactory validation evidence in patients who underwent hip arthroplasty.

## INTRODUCTION

 The functional outcomes of hip or knee arthroplasty can be assessed using health-related quality of life (HRQOL) instruments, including questionnaires and scales. The instruments available in the current literature evaluate pain as the principal symptom, which presents a limiting factor in the performance of low-demand activities of daily living (ADL).^1^

 This emphasis on pain and ADL gives rise to a degree of difficulty when discerning individuals who demonstrate no pain limitations for low-demand activities such as ADL, but who endure limitations when participating in more demanding activities such as sports. Assessing important functional differences (e.g., walking on uneven ground, running, climbing stairs, and level of physical or sporting performance) is not possible with currently available instruments.^2^

 In response, Talbot et al. developed and validated the High-Activity Arthroplasty Score (HAAS). This was designed to assess the physical function of patients after lower limb arthroplasty (hip and/or knee), incorporating a greater spectrum of physical and sporting activities, in addition to the customary emphasis on painful symptoms. It is a 4-item self-administered instrument in a scalogram format designed to assess physical function with items addressing (i) *Walking*, (ii) *Running*, (iii) *Stair Climbing*, and (iv) *Activity Level*. Each item assessed the highest capacity of the patient, reflecting a total score ranging from 0 to 18. Higher scores signify greater patient function.^2^ The HAAS was developed in the British English language and then cross-culturally adapted into the Brazilian Portuguese language, that is, HAAS-Brazil. Psychometric properties were at the time, yet to be evaluated within the Brazilian population.^3^

## OBJECTIVE

 This study aimed to investigate the validity of the psychometric properties of the scores produced by the instrument in a sample of post-hip arthroplasty Brazilian patients. We hypothesized that HAAS-Brazil would present acceptable evidence of validity for this purpose. 

## METHODS

### Ethical aspects

 This study was approved by the Ethics Committee of Hospital Universitário Pedro Ernesto (number 50529321.3.0000.5259; August 30, 2021). All participants signed an informed consent statement through paper or online application in accordance with the Brazilian National Health Council 510^th^ Resolution of April 7^th^, 2016. 

### Study design

 This was a cross-sectional study of the quantitative-qualitative nature of the assessment of psychometric properties of scores produced by a self-administered instrument utilizing primary data collected between June 2023 and April 2024. 

### Construct definition and conceptual framework

 The HAAS aims to measure the construct of physical activity, defined as any bodily movement produced by skeletal muscles that results in energy expenditure, referring to all movements, whether during moments of leisure, commuting, or as part of an individual’s work, from the perspective of estimating the ability to perform certain activities through the subjectivity of self-report.^4,6^ The items of the HAAS are related to the construct with respect to the conceptual framework of a reflective model.^7^

### Instruments

 HAAS is a self-administered instrument designed to evaluate physical function after lower limb arthroplasty (hip and/or knee), with a focus on higher-demand physical activities, such as sports participation.^2^ HAAS contains four items organized as a scalogram.^7,8^ Each item possesses its individual point system based within a hierarchical order which in sum, results in a possible range of scores from 0 to 18 points. Higher scores indicated better functional ability.^2,3,8^

 The 12-Item Short-Form Health Survey (SF-12) is a short instrument for measuring general HRQOL and assessing both physical and mental health using two summary scores: a physical component summary (PCS-12) and a mental component summary (MCS-12). Higher scores indicated higher HRQOL.^9^

 The Hip Outcome Score (HOS) is a 28-item instrument divided into two domains: ADL (19 items) and sports (9 items). It was developed to assess function in patients with hip disorders who are young and/or physically active but do not have severe degenerative abnormalities. Only the sports domain (HOS-Sp) was factored in for hypothesis testing in this study.^10^

### Sample size, participants selection and data collection

 Sample size was estimated according to the COnsensus-based Standards for the selection of health status Measurement INstruments (COSMIN) methodology of 7 times the number of items with at least 100 participants.^11,12^ The selection considered a non-probabilistic convenience sampling of patients with at least 6 months after hip arthroplasty, American Society of Anesthesiologists (ASA) classification ≤ 2 by the time of elective surgery, and fluent Portuguese reading ability.^13^ Arthroplasty surgery for a hip fracture was an exclusion criterion. Data were collected in a self-administered manner either by paper and pen on follow-up consultation or by digital collection through Google Forms. 

### Psychometric properties and statistical analyses

#### Validity Domain

##### Content validity

 Content validity examines the extent to which the concepts of interest are comprehensively represented by the items in the questionnaire and is recognized as the most important property in a patient-related outcome measure (PROM).^11,12^ Content validity was partially assessed on cross-cultural adaptation through expert panel opinion and the three-step test interview (TSTI) technique applied to volunteers evaluating the instrument.^3^ This study aimed to assess relevance, comprehensiveness, and comprehensibleness in relation to the construct of interest. As such, the retest was administered using a 5-item Likert-scale per item for comprehension, an open-ended question, and three dichotomous questions about face validity, relevance, and comprehensibility in a subgroup of patients. The content validity coefficient (CVC) was employed to evaluate content validity evaluation.^14^

##### Structural validity

 The internal structure refers to how different items in the PROM are related. Structural validity refers to the degree to which PROM scores adequately reflect the dimensionality of the construct to be measured. It was assessed by applying Mokken scale analysis (MSA), which addresses the assumptions inherent within a scalogram: 1) unidimensionality, 2) independence of item scoring, 3) monotonicity, and 4) invariant item ordering (IIO).^11,15-18^ These assumptions were investigated using the automated item selection procedure (AISP), scalability coefficient analysis (H), and evaluation of item response function.^16,19,20^

##### Construct validity

 Construct validity refers to the degree to which the scores of a PROM are consistent with hypotheses testing regarding its relationship with the scores of other instruments.^11,21^ This study correlated HAAS scores with HOS-Sp, PSC-12, and MSC-12. 

 A positive correlation was expected between HAAS and HOS-Sp scores. In comparison with SF-12, a positive correlation was expected in PSC-12 while in MSC-12 this was not have been observed. Correlation was measured by Spearman test (ρ). Correlations with HOS-Sp and PSC-12 should be ≥ 0.50, with higher magnitude in HOS-Sp due to the similarity of the construct of measurement. Correlation with MSC-12 should be lower, i.e., 0.30 – 0.50, since it measures related, but dissimilar constructs.^11,15^

##### Ceiling and floor effect

 Ceiling and floor effects are types of scale attenuation effects that are levels above or below variance, respectively, when an independent variable is no longer measurable. These effects are data gathering, which clusters responses, thereby compromising the ability to distinguish outcomes of patients on the highest and lowest levels of the instrument score.^22,23^ Ceiling and floor effects contribute to measurement inaccuracy.^22,23^ The presence of ceiling and floor effects was considered if there was clustering of > 15% of the maximum or minimum scores throughout the sample.^24^

#### Reliability Domain

##### Internal consistency

 Internal consistency refers to the degree of interrelatedness among the items and will be assessed by Cronbach α and Mokken scale ρ coefficients.^25,26^ Coefficient levels between 0.70 and 0.95 indicates a good internal consistency.^11^

##### Test-retest reliability

 The test-retest reliability evaluates the extent to which scores for patients who exhibit no change in their clinical status and who, over time, remain the same for repeated measurement.^27^ To evaluate the agreement between the test and a subsequent retest with a 48-hour to 3-week interval, the intraclass correlation coefficient (two-way mixed effects with average mean of measurements and absolute agreement ICC_(3,K)_) was applied. By assuming no normality of scores, the Wilcoxon signed-rank test evaluates the null hypotheses of no difference between the mean scores of the two applications.^11,28^

### Statistical analysis

 Statistical analysis was performed using IBM SPSS Statistics for Windows, Version 28.0 (IBM, Armonk, New York, United States) with a level of significance of 5% and R programming with the Mokken package.^29,30^

## RESULTS

 Patients were invited to participate in the research either in person or via cell phone text messaging. Consent and adequate completion of the test were obtained from 112 participants, while 81 patients answered the retest. A subgroup of 26 patients completed a re-test for content validity evaluation. Patient characteristics are summarized in Table 1. 

**Table 1:** Descriptive data of patients

**Variables**	**Values**
Sex, n (%)	
Male	55 (49.1%)
Female	57 (50.9%)
Age, years	55.92 ± 14.43
Weight, kg	77.43 ± 16.35
Height, m	1.68 ± 0.09
BMI*, kg/m^2^	27.37 ± 4.85
Underweight, n (%)	2 (1.8%)
Eutrophic, n (%)	33 (29.5%)
Overweight, n (%)	50 (44.6%)
Obese, n (%)	27 (24.1%)
Follow up, months	33.19 ± 30.11
Minimum	6
Maximum	141
Physical activity, n (%)	
Yes	96 (85.7%)
No	15 (13.4%)
HAAS	
Test (n = 112)	9.58 ± 3.72
Retest (n = 81)	10.48 ± 3.47
HOS-Sp	74.41% ± 27.71%
SF-12	
PSC-12	49.49 ± 9.27
MSC-12	52.42 ± 9.66

BMI = body mass index; HAAS = High Activity Arthroplasty Score; HOS-Sp = Sports domain of Hip Outcome Score; SF-12 = 12-Item Short-Form Health Survey; PSC-12 = physical domain; MSC-12 = mental domain;

* BMI categories according to *Ministério da Saúde* (Brazil).

### Content validity

 Content validity was assessed on cross-cultural adaptation by means of an expert panel opinion and the TSTI technique applied to volunteers from a diverse socio-educational sample who evaluated the translated instrument.^3^ To assess relevance, comprehensiveness, and comprehensibility among patients the CVC for each item was calculated (Table 2). Furthermore, all respondents answered affirmatively to question (1) "*Knowing that the aim of the questionnaire above is to evaluate the level of physical activity of patients after total hip replacement surgery, do you think it accomplishes its purpose?*" and (2) "*Do you think the questions of HAAS-Brazil are relevant to its purpose?*". 

**Table 2:** Content validity coefficient results

**Items**	**CVC**
*Walking*	0.931
*Running*	0.977
*Climbing stairs*	0.969
*Physical Activity*	0.946
Total	0.956

CVC = content validity coefficient.

### Structural validity

 The AISP investigation attested to a 4-item single cluster, reflecting the unidimensionality of the scale. No violations of monotonicity or IIO were observed in accordance with the MSA assumptions (Table 3). 

**Table 3:** Mokken Scale Analysis results

	**Cluster**	**H_i_ (SE)**	**VI_Mon_**	**P value**	**VI_IIO_**	**P value**
*Walking*	1	0.446 (0.077)	0	< 0.001	0	< 0.001
*Running*	1	0.509 (0.060)	0	< 0.001	0	< 0.001
*Climbing stairs*	1	0.370 (0.088)	0	< 0.001	0	< 0.001
*Physical Activity*	1	0.356 (0.085)	0	< 0.001	0	< 0.001

H_i_ = item scalability coefficient; VI_Mon_ = number of violations of monotonicity; VI_IIO_ = number of violations of invariant item ordering; SE = standard error.

### Construct validity

 A positive correlation was observed between the HAAS and HOS-Sp scores and between the HAAS and PSC-12 scores. Meanwhile, MSC-12 scores showed a lower correlation with HAAS scores, as predicted by the initial hypothesis. These findings are summarized in Table 4. 

**Table 4:** Spearman’s test correlation (rhô) between scores

	**PSC-12**	**MSC-12**	**HOS-Sp**	**HAAS**
PSC-12	-			
MSC-12	-0.049	-		
HOS-Sp	0.607**	0.297	-	
HAAS	0.554**	0.338**	0.695**	-

PSC-12 = physical domain of short form 12; MSC-12 = mental domain of short form 12; HOS-Sp = Sports domain of Hip Outcome Score; HAAS = High Activity Arthroplasty Score;

** P < 0.001.

### Ceiling and floor effect

 HAAS scores presented no ceiling or floor effect since the maximum (18) and minimum (0) scores accounted for less than 15% of the scores, as shown by the percentile analysis (P15 = 6.65; P85 = 13.35). 

### Internal consistency

 Internal consistency was assessed by Cronbach α coefficient (α = 0.663) favoring a questionable consistency while Mokken scale ρ (ρ = 0.707) was acceptable. 

### Test-retest reliability

 The test-retest reliability was evaluated for 70 applications and deemed adequate (ICC_(3,K)_ = 0.840 [F(69) = 6.225, P < 0.001]). The normality assumption of the sample favored a normal distribution for test scores (W = 0.988, P = 0.737), but was asymmetrical for retest scores (W = 0.952, P < 0.009). The Wilcoxon signed rank test was therefore performed to evaluate the difference between the paired applications, showing no significant difference (Z = -1.209, P = 0.230). 

## DISCUSSION

 Validation evidence was investigated foremost from content validity, as recommended by COSMIN.^24^ This investigation preceded evidence validation based on internal structure and, ultimately, evidence based on external measures.^24^ Aiming to ensure that the items of the HAAS are relevant, comprehensive, and comprehensible with respect to the construct of interest. Content validity was assessed on the adaptation process by TSTI and CVC of respondents from various ages, socioeconomic, and scholarity levels.^3^ This study enhanced the evaluation of content from a subgroup of patients, with a satisfactory performance of the instrument (CVC > 0.90) (Table 2).^14^

 As HAAS-Brazil is a scalogram, its internal structure was evaluated by MSA, which is a model of non-parametric item response theory based on a non-parametric probabilistic version of scalogram.^16-19,30^ MSA commands respect to four basic assumptions: 1) unidimensionality – items must measure only one latent trait (θ); 2) local independence of items’ score – association between items is explained exclusively by θ; 3) monotonicity – higher θ levels imply greater probability of endorsement of higher difficulty items; and 4) IIO – the item response function to each item must not intercept.^16,17^ AISP was therefore performed to evaluate if items cluster in one or more dimensions, which confirmed the unidimensionality of HAAS (Table 3). 

 As an adequate unidimensional instrument, the scalability coefficient (H) was used to investigate the model adjustment of the data and the quality of single items and each pair of items (H_i_ and H_ij_, respectively). This analysis explored the efficacy of scalograms in mitigating Guttman errors. This demonstrates the instrument’s ability to rank patients accurately based on their total scale scores. When evaluated in pairs, items should demonstrate positive covariance (H_ij_ > 0). Moreover, the scalability coefficient should be H > 0.30. The HAAS-Brazil produced by the response of posthip arthroplasty patients attended to all assumptions of MSA.^16,20^

 The internal consistency of instruments can be evaluated by Cronbach α coefficient, being the most widely employed estimator of reliability. However, there is increasing concern in the psychometric literature regarding its use. Because Cronbach α is negatively biased, especially in shorter scales such as HAAS, this study also evaluated internal consistency using the Mokken scale ρ (ρ = 0.707). HAAS-Brazil showed adequate internal consistency.^26,31^

 After gathering satisfactory evidence of the internal structure validity of HAAS-Brazil, test-retest reliability was assessed using ICC_(3,K)_. While the Shapiro-Wilk test favored normality in the test score distribution (W = 0.988, P = 0.737), we opted to employ non-parametric tests, such as the Wilcoxon signed-rank test. Physical activity is a construct that is empirically not expected to behave normally in the general population.^31^ The retest was applied to 81 patients with an interval ranging from 5 to 122 days. Since the interval must provide an adequate timeline to prevent memory bias and avoid latent trait-level alteration due to clinical changes, a maximum cutoff of 21 days was adopted after expert deliberation.^11,14^ The stability of test scores upon retesting, as undertaken by 71 patients, was satisfactorily attested. (ICC_(3,K)_ = 0.840 [F(69) = 6.225, P < 0.001]; Z = -1.209, P = 0.230). 

 Construct validity, that is, evidence based on external measures, was evaluated by applying Spearman’s test in accordance with the hypothesis of correlation between instruments. The strong correlation between the HAAS and HOS-Sp is best explained by the shared construct of interest, even though the HAAS centers on a broader definition of physical activity than the HOS-Sp, which focuses on sports. As a general HRQOL instrument, the SF-12 summary components are expected to possess lower correlation magnitudes with HAAS, particularly the MSC-12. The quality of the construct validity investigation resides in sound hypothesis testing, which was accomplished in this study.^15^

 Responsiveness analysis was beyond the scope of this cross-sectional study and will be investigated in a subsequent prospective evaluation of post-hip arthroplasty patients. The assessment of psychometric properties in patients undergoing knee replacement is currently underway. 

## CONCLUSION

 In this study, the psychometric properties of the Brazilian version of HAAS (i.e., HAAS-Brazil), which was adapted by Oliveira et al., were evaluated.^3^ The most important finding was that the HAAS-Brazil of post-hip arthroplasty patients gathered sound evidence of validity and reliability. These results support the applicability of HAAS-Brazil in both clinical practice and research settings. 
